# Archaeal Production of Polyhydroxyalkanoate (PHA) Co- and Terpolyesters from Biodiesel Industry-Derived By-Products

**DOI:** 10.1155/2013/129268

**Published:** 2013-12-19

**Authors:** Carmen Hermann-Krauss, Martin Koller, Alexander Muhr, Hubert Fasl, Franz Stelzer, Gerhart Braunegg

**Affiliations:** ^1^Institute of Biotechnology and Biochemical Engineering, Graz University of Technology, Petersgasse 12, 8010 Graz, Austria; ^2^ARENA Arbeitsgemeinschaft für Ressourcenschonende und Nachhaltige Technologien, Inffeldgasse 23, 8010 Graz, Austria; ^3^Institute of Chemistry, Karl-Franzens University of Graz, Heinrichstrasse 28, 8010 Graz, Austria; ^4^Polymer Competence Center Leoben GmbH, c/o Petersgasse 12, 8010 Graz, Austria; ^5^Institute for Chemistry and Technology of Materials, Graz University of Technology, Stremayrgasse 9, 8010 Graz, Austria

## Abstract

The archaeon *Haloferax mediterranei* was selected for production of PHA co- and terpolyesters using inexpensive crude glycerol phase (CGP) from biodiesel production as carbon source. CGP was assessed by comparison with the application of pure glycerol. Applying pure glycerol, a copolyester with a molar fraction of 3-hydroxybutyrate (3HB) of 0.90 mol/mol and 3-hydroxyvalerate (3HV) of 0.10 mol/mol, was produced at a volumetric productivity of 0.12 g/Lh and an intracellular PHA content of 75.4 wt.-% in the sum of biomass protein plus PHA. Application of CGP resulted in the same polyester composition and volumetric productivity, indicating the feasibility of applying CGP as feedstock. Analysis of molar mass distribution revealed a weight average molar mass *M*_*w*_ of 150 kDa and polydispersity *P*_*i*_ of 2.1 for pure glycerol and 253 kDa and 2.7 for CGP, respectively; melting temperatures ranged between 130 and 140°C in both setups. Supplying **γ**-butyrolactone as 4-hydroxybutyrate (4HB) precursor resulted in a poly[(*R*)-3-hydroxybutyrate-*co*-(*R*)-3-hydroxyvalerate-co-4-hydroxybutyrate] (PHBHV4HB) terpolyester containing 3HV (0.12 mol/mol) and 4HB (0.05 mol/mol) in the poly[(*R*)-3-hydroxybutyrate] (PHB) matrix; in addition, this process runs without sterilization of the bioreactor. The terpolyester displayed reduced melting (melting endotherms at 122 and 137°C) and glass transition temperature (2.5°C), increased molar mass (391 kDa), and a polydispersity similar to the copolyesters.

## 1. Introduction

Polyhydroxyalkanoates (PHAs) attract increasing attention as biobased, biocompatible, and biodegradable “green plastics.” This is due to their promising material properties and sound integration of their life cycle into nature's closed carbon balance; neither their production nor their application or degradation causes negative ecological impacts [1]. These polyoxoesters of hydroxyalkanoic acids (HAs) constitute reserves for carbon and energy accumulated by various prokaryotic genera among eubacteria and archaea [2]. Factors generally supporting intracellular PHA biosynthesis are, similar to the accumulation of other microbial storage compounds such as glycogen, a high intracellular energy charge, characterized by high pools of acetyl-CoA, ATP, or NAD(P)H. Such conditions result from a sufficient supply of carbon substrates together with suboptimal availability of growth-determining components such as nitrogen, phosphate, or growth-essential micronutrients [1].

Depending on the provided carbon source and the microbial production strain, the material properties of PHA resemble those of crystalline thermoplasts to flexible elastomers, latexes, and even high-performance, functional polymers; hence, they can be used for further processing towards vendible items. After their life span, PHAs are completely biodegradable to CO_2_ and water as the final products of mineralization [3]. Their exact material properties highly depend on the composition on the monomeric level. Here, rather crystalline, thermoplastic short chain length PHAs (*scl*-PHAs), consisting of HAs with 3 to 5 carbon atoms, are distinguished from elastomeric medium chain length PHAs (*mcl*-PHAs) which constitute polyesters of HAs consisting of 6 to 14 carbon atoms. Hence, they exhibit the potential to replace various petrol-based competitors in several bulk and niche segments of the plastic market in the near future [4].

The implementation of “white biotechnology” for production of frequently used materials such as bioplastics can be regarded as really encouraging for sustainable industrial development, although, for a range of products, especially bulk materials, biotechnological production strategies still have not yet passed the threshold for economic feasibility. Particularly, the economics of PHA production is to a high extent, namely, up to 50% of the entire production costs, defined by costs met for the supply of raw materials, especially the carbon substrates. This can easily be understood considering the fact that PHA accumulation occurs under aerobic conditions, resulting in high losses of carbon substrate by intracellular respiration. Hence, mainly due to a considerable loss of carbon by CO_2_ formation and excretion of water soluble metabolites, only a maximum amount below half of the carbon source is directed towards cell biomass growth and PHA accumulation. Classically, PHAs are produced starting from prized substrates of high nutritional value, such as glucose, starch, or edible oils [5]. Similar to the contemporary “fuel versus food” or “tank versus plate” controversy, this causes severe ethical conflicts considering the huge number of persons starving worldwide. Application of substrates of nutritional value for production of biopolymers or biofuels has an unquestioned impact on food prices, acting as a driving force for inflation, thus making food nearby unattainable for numerous people. As an outdoor, PHAs can be produced starting from inexpensive renewable resources as carbon feedstocks. For this purpose, pure saccharides, lipids, agroindustrial waste like hydrolysis products of (ligno)cellulosics, starch, crude glycerol phase (CGP) from biodiesel production or glycerol pitch from petrochemistry, lipids, whey lactose from dairy industry, alcohols like methanol, methane from anaerobic digestion of organic waste, and CO_2_ from industrial effluents are available at sufficient quantities [6–9]. The utilization of waste materials as feedstocks for PHA biosynthesis constitutes a viable strategy for cost-efficient biopolymer production and supports various agro-industrial branches to overcome the existing waste disposal problems.

In the last years, a considerable increase in the production of biodiesel has caused a sharp decrease in the cost of glycerol, the main by-product of biodiesel manufacturing; about 10% of glycerol accrues considering the quantity of lipids used as feedstock [10, 11]. As a result, glycerol has become a very attractive substrate for “white biotechnology.” Additionally, because carbon atoms in glycerol are higher reduced than in carbohydrates, cells using glycerol are in a more reduced physiological state, favoring intracellular polymer synthesis. In general, glycerol can be regarded as one of the most favorable substrates for generation of the PHB precursor acetyl-CoA. CGP from the biodiesel production contains up to 60% of glycerol and various impurities such as water, methanol, hydroxide residues, salts, fatty acids, and esters [12]. As methanol constitutes a severe cell toxin for many microbial strains, the selection of an adequate production strain that tolerates a certain concentration of this alcohol is desired. This is the case for *Methylomonas extorquens*, an organism that, if cultivated in CGP, first converts methanol before utilizing glycerol as carbon substrate, as previously reported by Braunegg and colleagues [13].

The use of glycerol for microbial PHA synthesis has been analyzed in natural PHA producers, such as, among others, *Methylobacterium rhodesianum* and *Ralstonia eutropha* [14], *Methylomonas extorquens* [13], *Azohydromonas lata* DSM 1124 [15], several Pseudomonas strains like *Ps. putida* KT2442 [11], the recently described *Zobellella denitrificans* [16], *Cupriavidus necator* [17, 18], *Novosphingobium capsulatum* [19], and *Bacillus* sp. [20]. Glycerol has also been investigated as a substrate for PHB synthesis in recombinant *E. coli* carrying the PHB biosynthetic genes from *Streptomyces aureofaciens *[21], *Burkholderia cepacia* ATCC 17759 [22], the archaea *Haloarcula *sp. IRU1 [23], *Halomonas* sp. KM-1 [24], and *Haloferax mediterranei* DSM 1411 [25].

Beside a convenient inexpensive raw material, efficient PHA production requires stable, robust, and fast-growing microbial production strains. The archaeon *Haloferax mediterranei* was isolated at the Mediterranean coast and first exhaustively described as PHA producer by Rodriguez-Valera and Lillo [26]. Within the phylum of the Euryarchaeota, the organism belongs to the class of halobacteria, the extremely halophile branch of the domain Archaea. This strain not only tolerates high salinity, but even requires 2–5 M NaCl for growth. The genus *Haloferax* is of special interest due to the faster growth compared to related organisms, its high PHA-productivity, the high quality of PHA produced by the strain, and features a broader substrate spectrum; it is known to convert several mono-, di-, and polysaccharides [26, 27], *inter alia* whey-derived sugars, and several starch-rich feedstocks [28, 29]. In addition to biopolyesters, the strain is reported to produce a high-value extracellular polysaccharide (EPS) that might by applied in food technology due to its xanthan-like properties [2, 25]. This is well visible by the mucous character of *H. mediterranei* colonies growing on solid media; due to the high content in bacterioruberins, a group of C50 carotenoides, these colonies are pinkish to reddish shaded [30]. EPS production negatively impacts PHA biosynthesis due to the partial shift of carbon towards EPS instead of biopolyester production. Recently, it was demonstrated how to overcome this problem by knocking out part of the gene cluster responsible for EPS biosynthesis; compared to the wild type strain, this novel-engineered *H. mediterranei* strain featured enhanced PHA biosynthesis rate of about 20%. [31]. Only recently, the complete sequence of the *H. mediterranei* genome was deciphered and reported [32].

### 1.1. Aim of the Study

Based on the first reproducible results for archaeal PHA production on CGP [24], the presented work compares PHA copolyester biosynthesis on pure glycerol and CGP to biosynthesis of PHA terpolyesters using CGP together with the 4-hydroxybutyrate (4HB) precursor *γ*-butyrolactone (GBL). *Haloferax mediterranei* was selected as robust archaeal production strain. The work encompasses kinetic process analysis and a detailed characterization of the obtained material properties regarding thermoanalysis and molar mass distribution. The elaborated data are discussed in comparison with literature data for PHA production by *H. mediterranei* on glucose, whey, and starch-based materials and shall be further used for designing a pilot-scale production plant for semi-industrial archaeal PHA production from CGP.

## 2. Materials and Methods

### 2.1. Production Strain

A lyophilized culture of *Haloferax mediterranei* DSM 1411 was purchased from DSMZ culture collection (Braunschweig, Germany).

### 2.2. Raw Material

CGP from tallow-base biodiesel production was obtained from Argent Energy (UK) Limited, UK.

### 2.3. Media Composition and Cultivation Conditions

The strain was cultivated in a 10 L bioreactor (L 1523, BIOENGINEERING, Wald, Switzerland) for production of poly[(*R*)3-(hydroxybutyrate-*co*-(*R*)-3-hydroxyvalerate) (PHBHV) copolyesters or poly[(*R*)-3-hydroxybutyrate-*co*-(*R*)-3-hydroxyvalerate-*co*-4-hydroxybutyrate) (P(HBHV4HB).

The basic saline medium was composed as follows (g/L): NaCl, 150; MgCl_2_∗6H_2_O, 13.0; KCl, 4.00; CaCl_2_∗2H_2_O, 13.0; FeCl_3_, 0.0025; trace element solution SL6 [33], 1; MgSO_4_∗7H_2_O, 20.0; NaHCO_3_, 0.20; NaBr, 0.50; complex nitrogen source (yeast extract/casein peptone, 1 : 1), 5.0; carbon source (glycerol or CGP, resp.), 10 g/L. In the case of P(HBHV4HB) production (fermentation F3), *γ*-butyrolactone (GBL) was added stepwise by addition of a total of 14 small portions during the phase of predominant PHA biosynthesis in order to keep the actual GBL concentration in a range of approximately 0.5 g/L (starting from 58 h until 168 h of cultivation; entire addition of GBL: 60.5 mL).

Inoculum cultures were prepared in 1 L baffled shaking flasks containing 250 mL saline medium, starting with 10 g/L glycerol as sole carbon source and 5 g/L complex nitrogen source. The pH value was adjusted to 7.0, and the cultures were continuously shaken for 48 h at 37°C. Bioreactor fermentations were started with 10 g/L carbon source (pure glycerol or CGP) in the case of fermentations F1 and F2 and 2 g/L in the case of fermentation F3; 5 g/L complex nitrogen source (yeast extract/casein peptone [1 : 1]) for each fermentation setup. In order to achieve phosphate limiting conditions as the most efficient factor provoking PHA synthesis in *H. mediterranei* [25], the only phosphate source derived from phosphate content presents in yeast extract. Further additions of substrates were done when necessary to keep the concentration of glycerol in a range between 10 and 20 g/L. The experiments were carried out under controlled conditions at a pH value of 7.0, a temperature of 37°C, and oxygen tension of about 20% of air saturation controlled by the agitation speed of the stirrer and the aeration rate.

### 2.4. Determination of Glycerol and GBL

A HPLC equipment consisting of a thermostated Aminex HPX 87H column (80°C), a HP 7673 Controller, a JASCO 880-PU intelligent HPLC pump, and a BISCHOFF RI-Detector 8110 were used. The elution solvent was 0.005 M H_2_SO_4_, with a flow of 0.60 mL min^−1^. Pure glycerol and GBL were used as external standards.

### 2.5. Determination of PHA

PHA content in cells was analyzed by acidic methanolysis according to Braunegg's method [34]. The gas chromatographic analysis was performed with a 6850 Network GC System (Agilent Technologies), equipped with a 25 m × 0.32 mm × 0.52 *μ*m HP5 capillary column and a flame ionization detector (FID). Helium (Linde; purity = 4.6) was used as carrier gas with a split ratio of 1 : 5, hydrogen (Linde; purity = 5.0) and synthetic air (Linde; purity = “free of hydrocarbons”) as detector gases, and nitrogen (Linde; purity = 5.0) as auxiliary gas.

The following protocol was used: initial temperature: 50°C; rate 1: 15°C/min; final temperature 1: 60°C; rate 2: 2°C/min; final temperature 2: 80°C; final temperature 3: 300°C; final time 3: 5 min. *mcl*-PHA were determined as follows: initial temperature: 50°C; rate 1: 15°C/min; final temperature 1: 200°C; final time 1 : 10 min; rate 2 : 15°C/min; final temperature 2: 240°C; final time 2: 4 min final temperature 3: 300°C; final time 3: 5 min. The determination of all samples was done in duplicate. Methyl esters of PHA-building blocks were analyzed using a flame ionization detector (FID). Helium acted as carrier gas, the applied split ratio amounted to 1:5, 2 *µ*L were used as injection volume.

As reference materials, poly[(*R*)-3HB-*co*-15.6%-(*R*)-3HV] (BIOPOL, ICI, UK) and poly[(*R*)-3HB-*co*-11.2%-4HB] (GreenBioTM, Tianjin Green Bioscience & DSM, PR China) were used as PHA references; hexanoic acid acted as internal standard.

### 2.6. PHA Extraction

After the stop of the bioreactor cultivations, cells were *in situ* pasteurized, centrifuged, frozen, and lyophilized for 24 hours. Biomass was degreased overnight by a Soxhlet extraction with ethanol, air-dried, and subsequently Soxhlet extracted overnight with CHCl_3_; extracted polymer and remaining biomass were finally analyzed by GC to determine the purity and the completeness of the extraction.

### 2.7. Determination of Molar Mass Distribution and Thermal Analysis Characterization

Molar masses, expressed as weight average molar mass *M*_*w*_ and number average molar mass *M*_*n*_, were measured on a Jasco PU-1580 HPLC connected to a Jasco 830-RI detector and equipped with two PLgel 5 *μ*m mixed C columns, with CHCl_3_ as solvent at a flow rate of 1.0 mL/min. Monodisperse polystyrene was used as external standard. Polydispersity *P*_*i*_ was defined as the ratio of *M*_*w*_ to *M*_*n*_.

Thermal analysis characterization was performed on a Mettler TA 4000 System instrument consisting of DSC-30 Differential Scanning Calorimeter, TGA-50 furnace with M3 microbalance, and TA72 GraphWare software. Measurements were carried out at 80 mL min^−1^ nitrogen of flow rate according to the following protocol: first, second, and third heating from −30 to 200°C at 10°C min^−1^ first cooling (quenching after the first heating) from 200 to −30°C at 100°C min^−1^, and the second cooling from 200 to −30°C at 10°C min^−1^.

### 2.8. Determination of Proteins

After disrupting cells by ultrasonic treatment, proteins were monitored according to Lowry's method [35].

## 3. Results and Discussion

### 3.1. F1: Production of P(HBHV) from Pure Glycerol

**Figure 1:**
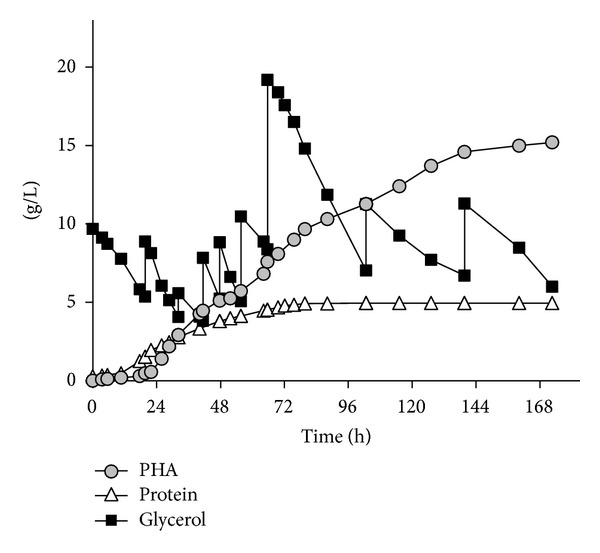
Fermentation pattern for PHA production on pure glycerol: time curves of substrate and products.

As single carbon source pure glycerol (10 g/L) was used, yeast extract/casein [1 : 1] (5 g/L) acted as complex nitrogen and phosphate source. Preexperiments performed by the authors of this study demonstrated that an exceeding of the substrate concentration beyond 20 g/L does not show any negative impact due to the high osmophilicity of the organism. Glycerol concentration during the fermentation therefore was kept between 10 and approximately 20 g/L. The time courses of glycerol as well as the concentrations of PHA and residual biomass (expressed as protein) are illustrated by the fermentation pattern in Figure 1. At the end of the fermentation, a maximum PHA concentration of 15.2 g/L and a protein concentration of 5.0 g/L were reached, corresponding to a maximum content of PHA in biomass (expressed as sum of protein and PHA) of 75%. Kinetic data are shown in Table 1, comparing the results of fermentation F1 with the other accomplished experiments and data from the literature using different main carbon sources. Due to the fact that specific production rate *π*_max⁡_ in the early production phase clearly differs from *π*_max⁡_ in the late production phase, *π*_max⁡,1_ and *π*_max⁡,2_ were introduced to describe this phenomenon. Here, *π*_max⁡,1_ amounted to 0.03 g/gh, whereas *π*_max⁡,2_ was calculated with 0.02 g/gh, respectively.

The volumetric productivity for PHA amounted to 0.12 g/Lh for the entire process that is drastically lower than reports on glucose [29] but higher than the valued reported for whey-stemming substrates [33, 36]. The highest productivity was observed between *t* = 26 and 80 h (0.21 g/Lh). Until the end of the process (*t* = 80–172 h), productivity significantly decreased to 0.06 g/Lh. The yield for conversion of glycerol towards PHA [*Y*_(PHA/substrate)_] amounted to 0.37 g/g that is very similar to data for semi-industrial eubacterial PHA production from hydrolyzed cane sugar (0.33 g/g) [37] and significantly higher if compared to reported PHA production on glucose and inorganic nitrogen and phosphate sources (0.26 g/g) [26].

The investigated organism is able to directly produce 3-hydroxyvalerate (3HV) units from glycerol, resulting in the accumulation of PHBHV containing about 10 mol-% of 3HV. In most cases, 3HV production requires the application of structurally related precursor substrates like propionic acid or valeric acid; these precursors significantly raise the production costs. In the case of *H. mediterranei*, the percentage of 3-HV in the polyester remained at a constant level of 10 mol-% during the production phase. This rare copolyester production from unrelated substrates can be explained by the particularities of the gene cluster (*phaEC*_*Hme*_) encoding *H. mediterranei* PHA synthase. Lu and colleagues revealed that in *H. mediterranei* a novel member of the class III PHA synthases, composed of *PhaC*_*Hme*_ and *PhaE*_*Hme*_, accounts for PHBHV synthesis [38]. More recently, Han and colleagues discovered that strain uses multiple pathways for propionyl-CoA biosynthesis as precursor for 3HV biosynthesis, including the citramalate/2-oxobutyrate pathway, the aspartate/2-oxobutyrate pathway, the methylmalonyl-CoA pathway, and a novel 3-hydroxypropionate pathway [39]. The metabolic pathways supplying the required intracellular precursors for PHBHV production by *H. mediterranei* were first investigated and described in detail by Feng and coworkers [40]. Additional investigation by whole-genome analysis has revealed eight potential *β*-ketothiolase genes in *H*. *mediterranei*, among which the PHBV-specific BktB and PhaA were identified by gene knockout and complementation analysis [41].

For most applications, a 3-HV share of at least 10 to more than 20 mol-% is necessary in order to sufficiently lower crystallinity of the product [42] and thus facilitate polyester processing. The formation of considerable shares of 3HV without the need for precursor substrates contributes to lowering the production costs of high-quality biopolyesters.

Molar mass distribution was determined at the end of the fermentation with a *weight average molecular weight* *M*_*w*_ = 150 kD and a polydispersity index of *P*_*i*_ of 2.1. The received molar mass is lower than reported molar masses for polyesters produced, for example, from carbohydrates like glucose [28] or whey lactose [33]. Madden and coworkers have proven that substances like glycerol or glycols cause a termination of chain propagation by covalent linking at the carboxyl terminus of PHA “endcapping effect”, resulting in lower molar masses [43]. Thermoanalysis of the polymer revealed a glass transition temperature *T*_*g*_ of 4.3°C, a cold crystallization point *T*_*c*_ at 67.2°C, and two melting endotherms *T*_*m*1_ and *T*_*m*2_ at 130.2 and 140.6°C. The appearance of multiple melting endotherms is a typical finding for *H. mediterranei* PHA and is also reported for PHA production on other carbon substrates like whey or glucose [25, 28]. By fractionation using chloroform/acetone, Don and colleagues revealed that the produced PHBHV consists of two copolymers of different composition, different melting points, and different molar masses [44].

### 3.2. F2: Production of PHBHV from Glycerol Liquid Phase (CGP) from Biodiesel Production

**Figure 2:**
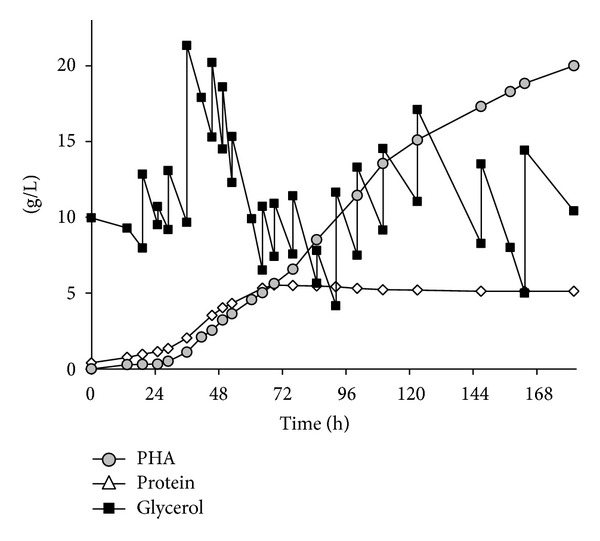
Fermentation pattern for PHA production on CGP: time curves of substrate and products.

In this case, pure glycerol was replaced by CGP as sole carbon source.  Figure 2 shows the fermentation pattern, including time curves for glycerol, PHA, and protein.

A comparison of the results with the previous fermentation F1 reveals a specific growth rate *μ*_max⁡_ of 0.06 h^−1^, significantly lower than on pure glycerol (0.10 h^−1^). This might be due to inhibiting compounds in GGP remaining from the transesterification process, such as methanol or lipid residues. Moreover, specific production rate *π*_max⁡1_ in the early production phase was slightly lower than that on pure glycerol, whereas specific production rates *π*_max⁡2_ in the late production phase were equal (see Table 1). The yield for PHA conversion towards PHA [*Y*_(PHA/substrate)_] (0.19 g/g) was lower than that for pure glycerol (0.37 g/g). Looking at the final concentration for protein (5.5 g/L versus 5.0 g/L) and PHA (16.2 g/L versus 13.4 g/L), significantly higher concentrations was obtained using CGP than in the case of pure glycerol; volumetric productivity for the entire process were identical for both processes(0.12 g/Lh; Table 1). The highest productivities were observed between *t* = 29 and 147 h (0.16 g/Lh); until the end of the process, productivity decreased to 0.03 g/Lh.

The composition of the copolyester produced from CGP was similar to the one produced from pure glycerol (F1) and to the literature data for glucose [28] and whey lactose [33]. Also here, the strain was able to produce 3-HV units for P(HBHV) copolyester synthesis with a molar 3-HV fraction in the copolyester of 0.10. Molar mass and molar mass distribution were determined at the end of the fermentation with *M*_*w*_ = 253 kDa and *P*_*i*_ = 2.7. Similar to the values for the F1 polyester, thermoanalysis of the polymer showed a glass transition temperature *T*_*g*_ of  7.0°C, a cold crystallization point *T*_*c*_ at 64.5°C, and two melting endotherms *T*_*m*1_ and *T*_*m*2_ at 128.7 and 138.8°C (see Table 1).

### 3.3. F3: Production of P(HBHV4HB) from CGP and GBL

In order to further improve the thermomechanical polyester properties, it was intended to produce a PHBHV4HB terpolyester consisting of the building blocks 3HB, 3HV, and 4HB. CGP was used as main carbon source for production of biomass, 3HB and 3HV, and GBL acted as precursor for 4HB production. This is a similar approach to the, until today, only reported archaeal terpolyester production by *H. mediterranei* on hydrolyzed whey lactose plus GBL [33]. According to literature reports, PHA terpolyesters harboring 4HB building blocks display advantageous properties if compared to PHBHV copolyesters, especially in terms of reduced secondary crystallization (typical of PHB and PHBHV) and increased elongation to break and tensile strength [45, 46]. GBL was added stepwise as cosubstrate after the end of the exponential growth phase (after 58 hours of cultivation; see Materials and Methods) to reach approximately 5% 4-HB in the polymer; finally, a terpolyester poly-[(*R*)-3HB-*co*-10%-(*R*)-3HV-*co*-5%-4HB) was obtained. Furthermore, this fermentation was run at reduced sterility precautions (no sterilization of the bioreactor equipment; only media components were heat sterilized in autoclaves) to demonstrate the high robustness of the applied production strain against contamination by microbial competitors. During the entire fermentation period, the culture maintained monoseptic, as surveyed microscopically. This is due to the high salinity of the cultivation medium counteracting the growth of most microbes. Considering the fact that microbial contamination is one of the major technical risks for continuous biotechnological processes, *H. mediterranei* appears especially suitable for such process mode, as already suggested previously [26].

**Figure 3:**
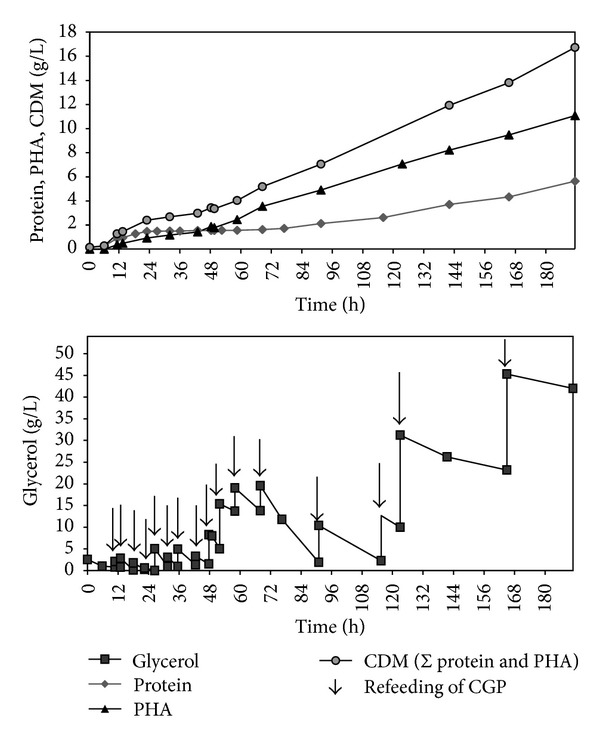
Concentrations of the substrate and the products during fermentation. Arrows indicate the re-feeding of substrate.

The initial concentration of glycerol was 2 g/L; additional CGP was added by feed pulses in order to maintain this concentration. After the stop of growth phase (48 h), the glycerol concentration was increased up to 15 g/L (see Figure 3). Yeast extract and casein hydrolysate (in a 1 : 1 ratio) were added after 6, 18, 26, 31.5, 35.5, 42.5, 52, 68, and 76.5 hours.

Figure 3 illustrates the glycerol concentration, protein production, cell dry mass (expressed as sum of protein and PHA), and polymer production. After a quite short lag phase, the exponential growth started after 6 hours. After 24 hours a protein content of 1.5 g/L was obtained; subsequently, the time curve of protein became linear. During the phase of exponential growth, a max. specific growth rate *μ*_max⁡._ of 0.20 h^−1^ was observed that constitutes the highest value of the investigated setups. This indicates that low starting concentrations of glycerol are beneficial for a short lag phase and fast cell growth. After the final addition of complex nitrogen and phosphate source after 76.5 hours, protein concentration increased again. Polymer production and the percentage of polymer in CDM started to increase at an early stage after 12 hours, reaching a level of 68.5% PHA in CDM after 68 hours, and maintained this amount until the end. A final protein concentration of 5.6 g/L was obtained at the end of fermentation (191 h), while the final polymer concentration amounted to 11.1 g/L. The volumetric productivity was calculated with 0.10 g/Lh considering the entire fermentation process which is slightly lower if compared to the prior PHBHV productions and terpolyester production from whey and GBL [33]. The highest productivity was observed between 58 and 191 h of cultivation; during the early stage of the fermentation (*t* = 0 to 58 h), only low productivity was observed (0.07 g/Lh) due to the restricted supply with carbon source during this phase that favored protein formation. The yield *Y*_(PHA/Glycerol)_ amounted to 0.16 g/g lower than it was the case for copolyester production and literature reports (see Table 1).

**Figure 4:**
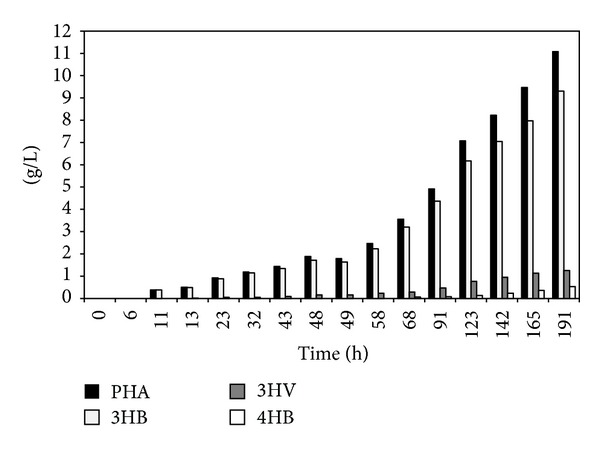
Formation of polyhydroxyalkanoate during fermentation.

GBL was first added continuously in order to obtain a homogenous distribution of 4HB in the terpolyester; GBL addition was accomplished from 58 hours until the end of the process. At the end of fermentation after 191 hours, the concentrations of produced HA-building blocks amounted to 9.3 g/L 3HB, 1.3 g/L 3HV, and 0.5 g/L 4HB, respectively; Figure 4 reflects the composition of the polymer during fermentation. The molar fraction of 3HV in PHA reached a stable value of 0.09–0.12 mol/mol. 4HB building blocks were detected immediately after the start of GBL feeding; the molar share increased steadily to reach a final value of 0.05 mol/mol at the end of fermentation, almost identical to the value in the whey-stemming terpolyester [33].

Molar mass (*M*_*w*_ = 391 kDa; the highest value of the three described setups) and molar mass distribution (*P*_*i*_ = 2.6; similar to the values for the copolyesters produced in F1 and F2) were determined at the end of the fermentation. Thermoanalysis of the terpolyester by DSC revealed a glass transition temperature *T*_*g*_ of 2.5°C, a cold crystallization point *T*_*c*_ at 64.3°C, and two melting endotherms *T*_*m*1_ and *T*_*m*2_ at 122.4 and 137.1°C. The start of polymer decomposition was 285°C. This is similar to the value obtained for the copolyester in F2 but significantly higher than that for the copolyester obtained from pure glycerol in the setup F1 (about 240°C). Comparing the data with the copolyesters from F1 and F2, the terpolyester showed a lower glass transition temperature and a decomposition point about 5°C higher.

## 4. Conclusion 

The presented experimental results demonstrate that expensive carbon sources for archaeal PHA production can be replaced by the inexpensive surplus product CGP from the biodiesel production process. Comparing CGP to pure glycerol phase does not reveal any negative effect in terms of productivity or polyester properties. The applied production strain *H. mediterranei* features the advantage to utilize inexpensive surplus products of industrial origin, lower energy requirements due to negligible sterility precautions, and the production of 3HV-containing copolyesters from unrelated carbon sources like glycerol. Further, similar to the application of sugars from hydrolyzed whey as main carbon source, the organism is able to accumulate PHBHV4HB terpolyesters when provided with adequate 4HB precursors.

Future work should focus on the production of the presented polymers on a larger scale for detailed assessment of their biodegradability, processability, and applicability for production of biobased blends and composites with other biocompatible materials. In addition, simple and convenient methods for recovery of PHA from the archaeal biomass have to be optimized for large-scale application; here, one can profit from the high intracellular pressure of *H. mediterranei* cells, disrupting them by exposure to hypotonic media (deionized water), releasing the biopolyester granules into the aqueous media. Regarding the process design, continuous production mode should be assessed based on first attempts described in the literature using this organism. Continuous mode should result in increased productivities during extended time periods and constant and reproducible product qualities. Especially, the tailored distribution of 4HB building blocks in PHBHV4HB can be facilitated by an optimized continuous feeding strategy. Finally, the presented results from laboratory scale are considered for planning and dimensioning the facilities and unit operations for semi-industrial archaeal PHA production from CGP waste stream of the biodiesel production. In order to save expenses for transportation of the raw material CGP, this production plant should be directly integrated into the existing production lines for biodiesel, where CGP directly accrues.

## Figures and Tables

**Table 1:** Maximum specific rates, yields, polymer productivity, and polymer characterization of different fermentations of *H. mediterranei*.

Maximum specific rates	F1^a^	F2^b^	F3^c^	PHBHV4HB from whey and *γ*-butyrolactone [32]^d^	PHBHV from whey [32]^d^	PHBHV from rice bran [29]^e^	PHBHV from glucose [28]^f^	PHBHV from glucose [26]^g^
*µ*_max⁡_[1/h]	0.10	0.06	0.20	0.14	0.10	0.098	0.09	~0.10
*π*_max,1_ [g/gh]; early prod. Phase	0.03	0.04	0.08	0.23	0.15	n.r.	n.r.	0.06
*π*_max,2 _ [g/gh]; late prod. Phase	0.02	0.02	0.01	0.01	0.11	n.r.	n.r.	~−0.006
Yields								
Y (PHA/carbon source) [g/g]	0.37	0.19	0.16	0.20	0.29	n.r.	n.r.	0.26
max. protein [g/L]	5.0	5.5	5.6	2.10	4.60	65.1	44.1	3.61
max. PHA [g/L]	13.4	16.2	11.1	14.7	12.20	77.8	41.7	5.13
PHA/CDM [%] (end)	—	—	—	—	—	55.6	48.6	58.7
PHA (PHA + biomass protein) [%] (end)	75.0	76.0	66.2	87.5	72.8	—	—	—
Productivity								
Vol. productivity [g/Lh] (PHA)	0.12	0.12	0.10	0.14	0.11	0.65	0.36	~0.08^i^; ~0.15^j^
Polymer characterization								
1st. Melting endotherm (*T*_*m*1_), [°C]	130.2	128.7	122.4	139.0	150.8	n.r.	134.8/131.2^k^	n.r.
2nd. Melting endotherm (*T*_*m*2_), [°C]	140.6	138.8	137.1	140.0	158.9	n.r.	144.4/140.7^k^	n.r.
Cold crystallization peak [°C]	67.2	64.5	64.3	n.r.	62.2	n.r.	64.0/−50.0^k^	n.r.
Glass transition temperature [°C]	4.3	7.0	2.5	−2.0	6.0	n.r.	1.56/−0.68^k^	n.r.
Onset of decomposition (*T*_*d*_) [°C]	~240	~280	~285	236	241	n.r.	n.r.	n.r.
3-HV/PHA [mol-%]	10	10	11-12	21.8	6–9	n.r.	10.7/12.3^k^	n.r.
4-HB/PHA [mol-%]	—	—	5	5.1	—	n.r.	—	n.r.
*M*_*w*_ [kDa]	150	253	391	986	1057	n.r.	570/72^k^	n.r.
Polydispersity index *P*_*i*_ (*M*_*w*_/*M*_*n*_)	2.1	2.7	2.6	1.5	1.5	n.r.	1.22/1.44^k^	n.r.

^a^Production of P(HBHV) from pure glycerol.

^b^Production of P(HBHV) from crude glycerol phase (CGP) from biodiesel production.

^c^Production of PHBHV4HB from CGP and *γ*-butyrolactone.

^d^Production of PHBHV4HB from hydrolyzed whey lactose plus sodium valerate as additional 3HV precursor and *γ*-butyrolactone.

^e^ Production of P(HBHV) from extruded rice bran and extruded corn starch (1/8 g/g).

^f^Production of P(HBHV) from glucose.

^g^Under phosphate-limited conditions; polyester reported as “PHB;” according to today's knowledge: P(HBHV). Values with “~” refer to the fact that the authors of the present study estimated these values from the original data provided in Figure 1 of [26].

^h^degradation of PHA during late phase of fermentation due to glucose limitation (batch set-up).

^i^Entire process.

^j^Before glucose limitation.

^k^Two fractions of copolyester with different 3HV molar fractions were isolated.

n.r.: Not reported.

## Abbreviations

CGP: Crude glycerol phase
EPS: Extracellular polysaccharide
F1–F3: Fermentation 1–3
HA: Hydroxyalkanoate
*mcl*-PHA: Medium chain length PHA
*M*_*w*_: Weight average molar mass
*M*_*n*_: Number average molar mass
*μ*_max⁡._: Maximum specific growth rate
PHA: Polyhydroxyalkanoate
PHB: Poly[(*R*)-3-hydroxybutyrate]
PHBHV: Poly[(*R*)3-(hydroxybutyrate-*co*-(*R*)-3-hydroxyvalerate)
PHBHV4HB: Poly[(*R*)-3-hydroxybutyrate-*co*-(*R*)-3-hydroxyvalerate-*co*-4-hydroxybutyrate]
*P*_*i*_: Polydispersity index
*π*_max⁡._: Maximum specific product formation rate
*scl*-PHA: Short chain length PHA
*T*_*c*_: Cold crystallization temperature
*T*_*d*_: Onset temperature of decomposition
*T*_*m*_: Melting temperature
*Y*: Yield for conversion of substrate to PHA
3HB: (*R*)-3-hydroxybutyrate
3HV: (*R*)-3-hydroxyvalerate
4HB: 4-Hydroxybutyrate.
